# Zur Notwendigkeit einer Finanz- und Strukturreform der Pflegeversicherung

**DOI:** 10.1007/s00103-023-03695-3

**Published:** 2023-04-25

**Authors:** Heinz Rothgang

**Affiliations:** 1 SOCIUM Forschungszentrum Ungleichheit und Sozialpolitik, Forschungszentrum Ungleichheit und Sozialpolitik, Universität Bremen, Postfach 330440, 28334 Bremen, Deutschland; 2 Wissenschaftsschwerpunkt Gesundheitswissenschaften, Universität Bremen, Bremen, Deutschland

**Keywords:** Langzeitpflege, Pflegeversicherung, Reformen, Eigenanteile, Integriertes Pflegeversicherungssystem, Long-term care, Long-term care insurance, Reforms, Co-payments, Integrated long-term care insurance system

## Abstract

Bei Einführung der Pflegeversicherung im Jahr 1994 wurden Ausgestaltungsentscheidungen getroffen, die das System bis heute prägen. In diesem Diskussionsbeitrag werden 3 dieser Entscheidungen untersucht. Jeweils wird ein Bewertungsmaßstab formuliert, um die aktuelle Situation zu beurteilen. Bei negativer Beurteilung werden Reformoptionen diskutiert.

Die Ausgestaltung der Pflegeversicherung mit betraglich begrenzten Versicherungsleistungen und nach oben offenen Eigenanteilen hat zu Gesamteigenanteilen geführt, die von der Mehrheit der Heimbewohnenden nicht mehr aus dem laufenden Einkommen finanziert werden können. Um ihre ursprünglichen Ziele erfüllen zu können, müsste die Pflegeversicherung daher vom Kopf auf die Füße gestellt werden – durch eine absolute Begrenzung der Eigenanteile in Höhe und Dauer.

Auch das „duale Versicherungssystem“ aus einer sozialen Pflegeversicherung und einer privaten Pflegepflichtversicherung hat sich als „Geburtsfehler“ des Systems erwiesen. Da das Kollektiv der Privatversicherten über eine wesentlich günstigere Risikostruktur und höhere Durchschnittseinkommen verfügt, ist die vom Bundesverfassungsgericht geforderte „gleichmäßige Lastenverteilung“ bei der Finanzierung nicht gegeben. Zur Behebung dieser Ungleichbehandlung ist die Überführung des dualen Systems in eine integrierte Pflegeversicherung oder zumindest ein Risikostrukturausgleich zwischen beiden Zweigen zu fordern.

Dass die Pflegeversicherung als eigener Zweig der Sozialversicherung eingeführt wurde, lässt sich dagegen rechtfertigen. Allerdings wäre es zur Überwindung von Schnittstellenproblemen notwendig, die Finanzierungskompetenz für die geriatrische Rehabilitation bei der Pflegeversicherung und die für die medizinische Behandlungspflege in Pflegeheimen bei der Krankenversicherung anzusiedeln.

## Abstract (en)

When long-term care insurance was introduced in 1994, a number of conceptual decisions were made that continue to shape the system today. This discussion article examines three of these decisions. In each case, an evaluation standard is formulated against which the current situation is assessed. In the case of a negative assessment, reform options are discussed.

In combination with the lack of benefit adjustments, the design of long-term care insurance as a system with capped insurance benefits and unlimited co-payments has led to co-payment levels in nursing homes that the majority of residents cannot cover from their income. Therefore, in order to fulfill its original objectives, long-term care insurance would have be turned upside down – by imposing an absolute limit on the amount and duration of the individual co-payments.

The “dual insurance system” consisting of a social insurance for the majority and a private mandatory plan for a minority of the population has also proved to be a “birth defect” of the system. Since the group of privately insured persons has a much more favorable risk structure and higher average incomes, the “equal distribution of burdens” in financing required by the Federal Constitutional Court does not exist. To remedy this inequality, the dual system must be transformed into an integrated long-term care insurance system, or at least a risk structure equalization scheme between the two branches must be implemented.

The introduction of long-term care insurance as a separate branch of social insurance, however, can be justified. In order to overcome interface problems, it would nevertheless be necessary to place the financing competence for geriatric rehabilitation with long-term care insurance and that for medical treatment care in nursing homes with health insurance.

## Einleitung

Vor knapp einem halben Jahrhundert hat das Kuratorium Deutsche Altershilfe mit einem Gutachten den Anstoß für eine Debatte zur sozialpolitischen Absicherung des Pflegerisikos gegeben. In diesem wurde beklagt, dass Menschen mit einer „normalen“ Erwerbsbiographie im Alter zwar vor dem Krankheits-, nicht aber vor dem Pflegerisiko geschützt seien [1]. Im Jahr 1994 wurde daraufhin die Pflegeversicherung in Deutschland eingeführt, was als sozialpolitischer Meilenstein gewürdigt werden muss – zugleich wurden mit den damaligen Entscheidungen über die Ausgestaltung der Pflegeversicherung Probleme geschaffen, die die heutige Notwendigkeit weitreichender Finanz- und Strukturreformen begründen. 3 dieser Entscheidungen werden nachfolgend aufgegriffen – die Ausgestaltung der Pflegeversicherung:

1. als Teilleistungssystem,
2. als „duales Versicherungssystem“ von sozialer Pflegeversicherung und privater Pflegepflichtversicherung sowie
3. als von der Krankenversicherung getrenntes eigenes Versicherungssystem.

Eingegangen wird dabei jeweils auf den Bewertungsmaßstab und die Problemlage, die sich aus der Abweichung der tatsächlichen Situation von der angestrebten ergibt, auf die Evaluation bereits erfolgter Maßnahmen – insofern es dazu gekommen ist – und auf die vorliegenden Reformoptionen bzw. die verbleibenden Reformnotwendigkeiten.

Die Folgen der Konzeption als Teilleistungssystem werden dabei ausschließlich anhand der vollstationären Pflege diskutiert, da die Problematik hier besonders augenfällig ist. Auch in der ambulanten Pflege führen steigende Eigenanteile zu negativen Effekten – hier aber vor allem durch Verzicht oder Verringerung der Inanspruchnahme von formellen Pflegeleistungen mit entsprechenden Folgen für die Pflegequalität. Nicht behandelt werden kann hier aus Platzgründen auch ein vierter Geburtsfehler der Pflegeversicherung: die leistungs-, leistungserbringungs- und ordnungsrechtliche Segmentierung des Systems in ambulante und stationäre Versorgung und ihre innovationshemmende Wirkung, die durch eine sektorfreie Ausgestaltung des Systems vermieden werden könnte (vgl. hierzu [2]).

## Pflegeversicherung als Teilleistungsversicherung

Die Pflegeversicherung wurde als ein Teilleistungssystem gegründet und unterscheidet sich damit von der Krankenversicherung. Um diesen Teilleistungscharakter als Bewertungsmaßstab nutzen zu können, ist es allerdings notwendig, sich zunächst zu vergegenwärtigen, was damit gemeint ist.

### Bewertungsmaßstab.

Die Forderung nach einer sozialstaatlichen Absicherung des Pflegerisikos wurde seit Mitte der 1970er-Jahre damit begründet, dass es gelte, die pflegebedingte Sozialhilfeabhängigkeit zu beenden. Dass Menschen auch nach einem durchschnittlichen Erwerbsleben durch auftretende Pflegebedürftigkeit regelmäßig in die Sozialhilfe abrutschen und zu Almosenempfängern werden, wurde als eines modernen Sozialstaats unwürdig kritisiert [3, 4]. Dies hat sich im allgemeinen Teil der Gesetzesbegründung für das Pflege-Versicherungsgesetz (PflegeVG) entsprechend niedergeschlagen:

„Die Pflegeversicherung soll … bewirken, daß in der überwiegenden Zahl der Pflegebedürftigen nicht mehr auf Sozialhilfe angewiesen ist; wer sein Leben lang gearbeitet und eine durchschnittliche Rente erworben hat, soll wegen der Kosten der Pflegebedürftigkeit nicht zum Sozialamt gehen müssen“ (Gesetzesbegründung, PflegeVG‑E, S. 2, Grammatikfehler im Original).

Dazu soll die Pflegeversicherung die pflegebedingten Kosten vollständig übernehmen. Entsprechend heißt es in der Gesetzesbegründung:

„Die Pflegekasse … trägt … den pflegebedingten Aufwand für die im Einzelfall erforderlichen Leistungen der Grundpflege, der aktivierenden Pflege …“ (Gesetzesbegründung, PflegeVG‑E, S. 115).

Im ersten Bericht der Bundesregierung wurde dies noch einmal bestätigt:

„Die Pflegeversicherung … soll eine Grundversorgung sicherstellen, die im Regelfall ausreicht, die pflegebedingten Aufwendungen abzudecken“ [5, S. 8 ff.].

Wenn heute darauf verwiesen wird, dass die Pflegeversicherung als „Teilkaskoversicherung“ eingeführt wurde, wird meist der Eindruck erweckt, dass eine Eigenbeteiligung der Pflegebedürftigen an den Pflegekosten, insbesondere bei vollstationärer Pflege, intendiert war. Das ist allerdings falsch. In Anlehnung an den Dreiteilungsvorschlag der Arbeiterwohlfahrt (AWO) aus dem Jahr 1976 [6] werden die Heimkosten in 3 Komponenten zerlegt:

1. pflegebedingte Kosten,
2. Investitionskosten (z. B. Gebäudemieten, Anlagegüter) sowie
3. Kosten für Unterkunft und Verpflegung.

Die Pflegebedürftigen sollten dabei lediglich für die Kosten der Unterkunft und Verpflegung aufkommen, während die Investitionskosten von den Ländern und die pflegebedingten Kosten vollständig von der Pflegeversicherung getragen werden sollten.

### Entwicklung der Eigenanteile in der Heimpflege im Zeitverlauf.

Tatsächlich waren die Versicherungsleistungen 1996, als die Leistungen der stationären Pflege erstmals gewährt wurden, in der Regel ausreichend, um die pflegebedingten Kosten für Pflegebedürftige in Pflegestufe I und II vollständig abzudecken. Lediglich bei Pflegstufe III gab es in einigen Bundesländern Einrichtungen, deren Sätze höher lagen als die Leistungen der Pflegeversicherung. Ausdruck dieses Verhältnisses war auch, dass die Pflegeversicherungsleistungen bei Heimpflege ursprünglich mit „Bis-zu“-Beträgen bezeichnet waren. Sie waren nach oben doppelt gedeckelt, weil die maximalen Versicherungsleistungen die in Rechnung gestellten pflegebedingten Kosten in der Regel überstiegen haben.

**Figure 1:**
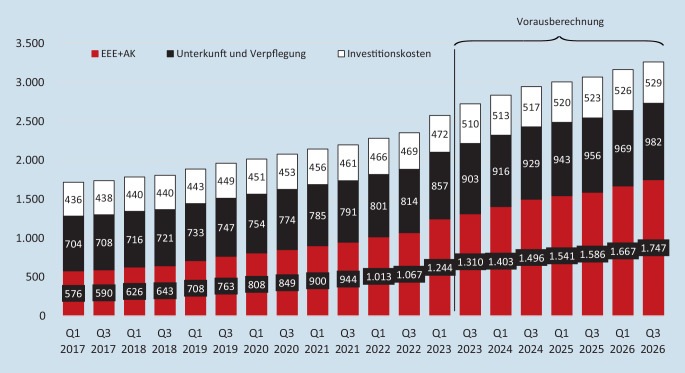

Bis 2008 sind die Leistungen der Pflegeversicherung dann nominal unverändert geblieben – im stationären Sektor in den Pflegestufen I und II, in die rund 80 % der Heimbewohnenden eingestuft wurden, sogar bis 2015. Da die Pflegesätze in dieser Zeit aber kontinuierlich gestiegen sind, hat sich der durchschnittliche in Bezug auf die Pflegegradverteilung gewogene Eigenanteil ausweislich der Pflegestatistik von 277 € Ende 1999 bis auf 602 € Ende 2015 erhöht. Das in den hier einschlägigen Teilen am 01.01.2017 in Kraft getreten Zweite Pflegestärkungsgesetz (PSG II) hat mit der Einführung der Pflegegrade und der entsprechenden Umstellung der Pflegesätze dann zwar zu einer kleinen Entlastung geführt, seitdem steigt der einrichtungseinheitliche Eigenanteil zu den Pflegekosten aber wieder kontinuierlich. Zudem sind die an die Pflegebedürftigen weitergeleiteten Ausbildungskosten (zur Vergütung von Auszubildenden, die in der Einrichtung beschäftigt sind) zu berücksichtigen, die inzwischen monatlich auch schon mehr als 100 € betragen. Wie Abb. 1 zeigt, beliefen sich die monatlichen pflegebedingten Eigenanteile (einschließlich Ausbildungskosten) bundesdurchschnittlich zum 01.01.2023 auf 1244 € und der bundesdurchschnittliche monatliche Gesamteigenanteil lag bei 2573 €, mehr als dem Doppelten der Durchschnittsrente für Männer. Auch in der Folgezeit ist mit einer weiteren Steigerung der Eigenanteile zu rechnen.

## Wirkungen des GVWG

**Figure 2:**
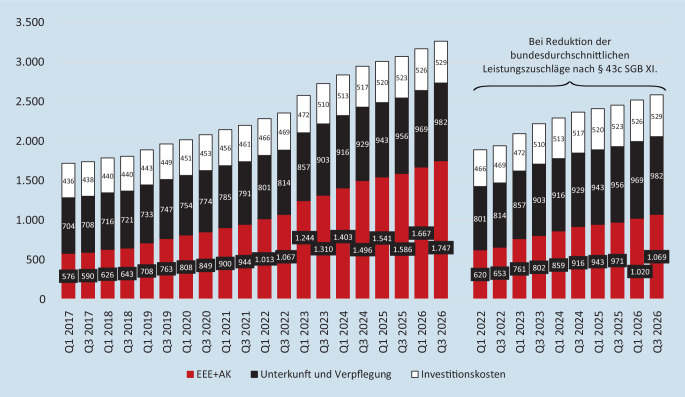

Zur Begrenzung des Eigenanteils an den pflegebedingten Aufwendungen der Heimpflege wurde im *Gesundheitsversorgungsweiterentwicklungsgesetz* (GVWG) vom 11.07.2021 (BGBl. I, S. 2754) ein Leistungszuschlag eingeführt, der je nach Dauer des Heimaufenthalts 5 % (bei einer Dauer von bis zu einem Jahr), 25 % (bei 1–2 Jahren), 45 % (bei 2–3 Jahren) bzw. 70 % (bei mehr als 3 Jahren) der pflegebedingten Eigenanteile (EEE + AK) beträgt (§ 43c SGB XI in der Fassung des GVWG). Tatsächlich sind die aufzubringenden durchschnittlichen Eigenanteile zum 01.01.2022 gesunken, steigen seitdem aber schneller als je zuvor wieder an (Abb. 2).

Seit September 2022 sind die Pflegeheime, die einen Versorgungsvertrag mit den Pflegekassen haben, verpflichtet, ihren Beschäftigten eine Entlohnung gemäß eines Tarifvertrags bzw. der entsprechenden kirchlichen Arbeitsrechtsregelung oder mindestens in Höhe eines einschlägigen Tarifvertrags bzw. der kirchlichen Regelung zu zahlen („Tariftreue“-Regelung). Dies hat gemeinsam mit der allgemeinen Kostensteigerung (z. B. aufgrund von Tariflohnsteigerungen) bis zum Jahresanfang 2023 zu einem bundesdurchschnittlichen Eigenanteil von 2573 € geführt. Werden hiervon durchschnittliche Leistungszuschläge von 483 € abgezogen, liegt der verbleibende durchschnittliche monatliche Eigenanteil zum 01.01.2023 bereits bei 2090 €, also knapp oberhalb des Niveaus des Eigenanteils von Mitte 2020. Weitere Steigerungen folgen aus der sukzessiven Umsetzung des Tariftreuegrundsatzes, aus der Einführung neuer und höherer Personalziffern zum 01.07.2023, der dann auftretenden Pflegesatzrelevanz von bisher zum Teil extern finanzierten Personalmengen und aus weiteren Umsetzungsschritten des Personalbemessungsverfahrens, wie sie im Referentenentwurf des Pflegeunterstützungs- und Entlastungsgesetzes vorgesehen sind.

**Figure 3:**
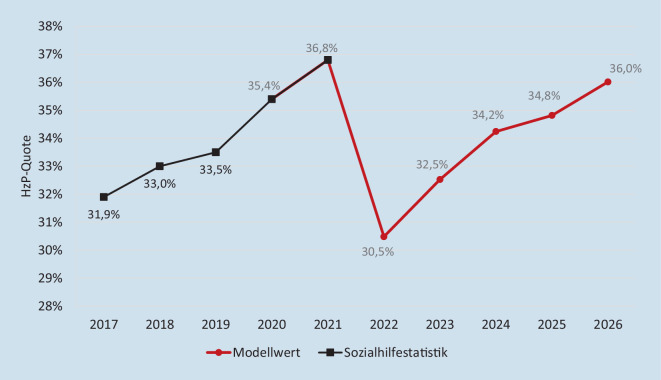

Entsprechend ist auch der Rückgang der Sozialhilfequote nur temporär. Aktuelle Modellrechnungen zeigen, dass der Anteil der Sozialhilfeempfänger:innen durch die Einführung der Leistungszuschläge zwar initial um 6,3 Prozentpunkte gesunken ist, dieser Effekt aber bereits nach 4 Jahren wieder aufgezehrt sein wird (Abb. 3).

### Verbleibende Reformnotwendigkeit.

**Figure 4:**
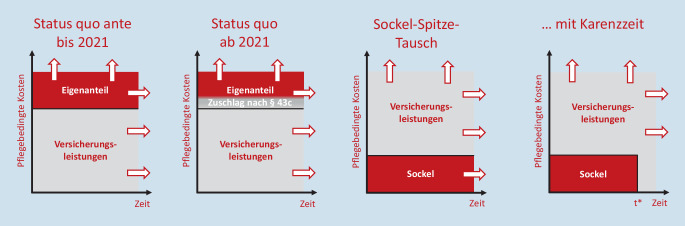

Um die pflegebedingten Eigenanteile in der Heimpflege nachdrücklich zu begrenzen, ist es notwendig, die Finanzierungsverantwortlichkeiten umzukehren. Abb. 4 stellt die damit angesprochenen Zusammenhänge schematisch dar. Seit ihrer Einführung zahlt die Pflegeversicherung einen gesetzlich fixierten festen Zuschuss zu den pflegebedingten Kosten. Kosten oberhalb dieses „Sockels“ müssen von den Pflegebedürftigen selbst aufgebracht werden – und zwar auf unbegrenzte Dauer („Status quo ante“). Mit dem GVWG übernimmt die Pflegeversicherung einen Teil des Eigenanteils, der von der Verteilung der Bezugsdauer vollstationärer Pflege abhängt. Derzeit liegt der von der Pflegeversicherung durch die Zuschläge übernommene Teil der Eigenanteile bei rund 38 %. Entsprechend sind 62 % der die Pflegeversicherungsleistungen übersteigenden pflegebedingten Kosten nach wie vor von der pflegebedürftigen Person zu tragen – und zwar wiederum zeitlich unbegrenzt („Status quo ab 2021“).

Beim von der Initiative „Pro-Pflegereform“^1^ initiierten Modell des „Sockel-Spitze-Tauschs“ zahlt dagegen die heimbewohnende Person einen festen Sockelbetrag, während die darüber hinausgehenden Entgelte vollständig von der Pflegeversicherung übernommen werden. Nur wenn die Sockelzahlung der pflegebedürftigen Person auf einen gegebenen Zeitraum begrenzt ist und der Sockelbetrag nach dieser Karenzzeit (in Abb. 4: t*) vollständig von der Pflegeversicherung übernommen wird („Sockel-Spitze-Tausch mit Karenzzeit“), ist der maximale Eigenanteil vorhersagbar, und ist eine zielgenaue Vorsorge möglich [2].

Die Effekte einer solchen Umstellung hängen natürlich von der Höhe des Sockelbetrags und der Dauer der Karenzzeit ab. Der damalige Bundesgesundheitsminister Jens Spahn hat in seinem Vorschlag vom Herbst 2020, der dann in einem Eckpunktepapier des Bundesgesundheitsministeriums präzisiert wurde, einen Sockelbetrag von monatlich 700 € und eine Karenzzeit von 36 Monaten bis zur vollständigen Übernahme der pflegebedingten Kosten durch die Pflegeversicherung empfohlen. Zudem sollten die Länder verpflichtet werden, pro belegten Heimplatz Investitionskosten in Höhe von monatlich 100 € zu übernehmen.

**Figure 5:**
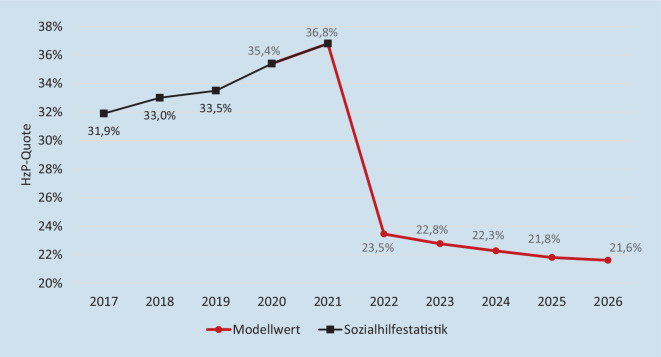

Gemäß der in Abb. 5 wiedergegebenen Modellrechnung hätte ein solche Parametrisierung des Modells des Sockel-Spitze-Tauschs bei Einführung im Jahr 2022 die Sozialhilfequote um mehr als 13 Prozentpunkte gesenkt – und dies nachhaltig. Allerdings führt dieses Modell im Vergleich zum Status quo (nach Inkrafttreten des GVWG) zu Mehrausgaben der Pflegeversicherung, die von 2 Mrd. € für 2022 auf knapp 6 Mrd. € für das Jahr 2026 anwachsen dürften (eigene Berechnungen mittels des in [7] erläuterten Simulationsmodells). Dem steht jedoch eine Verringerung der Sozialhilfeausgaben für Hilfe zur Pflege in Einrichtungen im Umfang von rund 1 Mrd. € (2022) bzw. 2,5 Mrd. € (2026) gegenüber. Das Modell des „Sockel-Spitze-Tauschs“ in der von Jens Spahn vorgeschlagenen Parametrisierung wäre damit in der Lage, nachhaltig zu verhindern, dass Pflegebedürftigkeit für einen Großteil der Bevölkerung zur Verarmung führt.

Auch eine Übernahme der Investitionskosten durch die Länder, wie sie bei Einführung der Pflegeversicherung geplant wurde, würde einen erheblichen Beitrag zur Reduktion der pflegebedingten Sozialhilfeabhängigkeit liefern können (Abb. 1). Schon die vom damaligen Gesundheitsminister Jens Spahn in seinen Eckpunkten geforderte sehr moderate Verpflichtung der Länder, einen zusätzlichen Investitionskostenzuschuss von 100 € pro belegtem Pflegeplatz bereitzustellen, ist aber im Verhandlungsprozess gescheitert – eine derartige Beteiligung der Länder erscheint in nächster Zeit kaum möglich.

### Steuerzuschüsse zur Pflegeversicherung.

Ein Sockel-Spitze-Tausch würde unter sonst gleichen Bedingungen zu einem Beitragssatzanstieg führen. Dieser könnte aber durch entsprechende Steuerzuschüsse vermieden werden. Steuerzuschüsse gibt es bereits in der gesetzlichen Renten- und Krankenversicherung. Sie wurden jeweils eingeführt, um die Ausgaben zu finanzieren, die der Sozialversicherung für die Erfüllung gesamtgesellschaftlicher Ausgaben erwachsen. Im Koalitionsvertrag wurde dazu vereinbart, „versicherungsfremde Leistungen wie die Rentenbeiträge für pflegende Angehörige und die pandemiebedingten Zusatzkosten aus Steuermitteln [zu] finanzieren“ [8]. Allein die Beiträge zur Rentenversicherung für pflegende Angehörige betrugen im Jahr 2021 3,07 Mrd. €. Ein Bundeszuschuss in entsprechender Höhe würde den Beitragssatz also um rund 0,2 Beitragssatzpunkte entlasten. Für die pandemiebedingten Zusatzkosten sind der sozialen Pflegeversicherung bis Ende des Jahres 2021 Ausgaben von mehr als 6 Mrd. € entstanden, die nicht aus Steuermitteln finanziert wurden [9]. Die Erfüllung des Koalitionsvertrags würde die Pflegeversicherung somit erheblich entlasten und die schon allein aus demografischen Gründen notwendigen Beitragssatzanstiege erheblich dämpfen.

## Duales System von Sozial- und Privatversicherung

Wie auch die Krankenversicherung ist die Pflegeversicherung „dual“ organisiert: Alle Mitglieder der gesetzlichen Krankenversicherung (GKV) unterliegen dabei der Versicherungspflicht in der sozialen Pflegeversicherung (SPV), während alle privat Krankenvollversicherten einen Versicherungsvertrag bei einem privaten Versicherungsunternehmen abschließen müssen, das als Träger der privaten Pflegepflichtversicherung (PPV) bei Pflegebedürftigkeit Leistungen in gleichem Umfang zur Verfügung stellt (§ 23 iVm § 110 SGB XI). Diese Zweiteilung ist Ergebnis der politischen Kompromissfindung bei Einführung der Pflegeversicherung [4] und ein weiterer Geburtsfehler der Pflegeversicherung, der Reformbedarf auslöst.

### Bewertungsmaßstab.

In seinem Urteil aus dem Jahr 2001 stellt das Bundesverfassungsgericht fest, dass der Gesetzgeber die Kompetenz hatte, eine „Pflegevolksversicherung in Gestalt zweier Versicherungszweige“ zu schaffen, bei der die Bevölkerung jeweils entweder der sozialen Pflegeversicherung oder der privaten Pflegepflichtversicherung zugewiesen wird. Allerdings sei diese Zuweisung nur unter der Maßgabe einer „ausgewogenen Lastenverteilung“ verfassungskonform ([10]: Rn 92). Von einer „ausgewogenen Lastenverteilung“ kann aber nur gesprochen werden, wenn die finanzielle Belastung für Versicherte in beiden Versicherungskollektiven in etwa gleich ist. Damit hat das Verfassungsgericht den normativen Bewertungsmaßstab geliefert, an dem die derzeitige Ausgestaltung der gesetzlichen (beide Versicherungszweige umfassenden) Pflegeversicherung bewertet werden kann und muss.

### Problemlage.

Tatsächlich liegt keine „ausgewogene Lastenverteilung“ vor: Erstens haben Privatversicherte eine günstigere Altersstruktur. Insbesondere bei den 80-Jährigen und Älteren, bei denen die Pflegehäufigkeiten besonders hoch sind, ist der Anteil der Privatversicherten deutlich niedriger als bei der Gesamtbevölkerung. Zweitens ist der Frauenanteil unter den Privatversicherten deutlich niedriger als in der Gesamtbevölkerung, was ausgabensenkend ist, da Frauen höhere Pflegekosten verursachen als Männer. Drittens sind die altersspezifischen Pflegehäufigkeiten für alle Altersklassen bei den Privatversicherten (teilweise um ein Vielfaches) niedriger als bei den Privatversicherten [11]. Im Ergebnis sind die Leistungsausgaben pro versicherte Person in der sozialen Pflegeversicherung – bei gleichlautenden Begutachtungsrichtlinien und gleichen Leistungen – rund 3‑mal so hoch wie in der privaten Pflegepflichtversicherung. Allerdings ist etwa die Hälfte der Privatversicherten beihilfeberechtigt. Werden die von der Beihilfe getragenen Ausgaben für PPV-Versicherte berücksichtigt, sind die Leistungsausgaben für eine sozialversicherte Person aber immer noch doppelt so hoch wie für eine privatversicherte (Tab. 1).

**Table 1:** 

	(1) Leistungsausgaben (in Mrd. Euro)	(2) Versicherte (in Mio.)	(3) = (1) / (2) Leistungsausgaben pro versicherte Person (in Euro)	(4) = (3_SPV_) / (3_PPV_) Zahlenverhältnis der jeweiligen Pro-Kopf-Ausgaben
*SPV*	50,200	73,51	682,90	–
*PPV*	2,071	9,19	225,39	3,03
*PPV zuzüglich Beihilfe*	3,107	9,19	338,08	2,02

*Anmerkung*: Rund die Hälfte aller Privatversicherten hat Beihilfeansprüche [12]. Die Höhe des Beihilfeanspruchs differiert zwischen den Bundesländern und dem Bund und nach Familienstand. Im Durchschnitt dürften aber rund 2/3 der Pflegekosten übernommen werden. Die von der PPV getragenen Leistungsausgaben betragen dann 0,5 × 1 + 0,5 × 1/3 = 2/3 der insgesamt von PPV und Beihilfe getragenen Ausgaben. Die Beihilfeausgaben für die Privatversicherten betragen 50 % der PPV-Ausgaben

*Abkürzungen*: *PPV* private Pflegepflichtversicherung, *SPV* soziale Pflegeversicherung

Unterschiede zeigen sich auch im jährlichen *Durchschnittseinkommen* der Versicherten. Nach Berechnungen in [15] beläuft sich dieses im Jahr 2016 bei den SPV-Versicherten auf 24.790 €, während es bei den PPV-Versicherten mit 52.287 € mehr als doppelt so hoch ist. Wird nur das nach den Regeln der GKV berechnete beitragspflichtige Einkommen der Privatvollversicherten betrachtet, verringert sich dieser Unterschied etwas, da die Beitragsbemessungsgrenze und die Beschränkung der beitragspflichtigen Einkommensarten bei Privatversicherten größere Wirkung zeigen als bei Sozialversicherten. Dennoch liegt das „beitragspflichtige Einkommen“ der Privatversicherten immer noch rund 2 Drittel über dem der SPV-Versicherten [15].

Unterschiede zwischen den beiden Versicherungszweigen zeigen sich somit sowohl bei den Einkommen als auch in der Risikostruktur. Der kombinierte Effekt zeigt sich, wenn der Beitragssatz berechnet wird, den das Kollektiv der bislang Privatversicherten zahlen müsste, wenn es eine eigene Sozialversicherung nach den Finanzierungsregeln der SPV bilden würde. Da die durchschnittlichen beitragspflichtigen Einkommen für das Privatversicherungskollektiv 2 Drittel höher liegen als für die Sozialversicherten, die Ausgaben aber um den Faktor 2,02 niedriger sind, läge der resultierende Beitragssatz um den Faktor 2,02 × 1,66 = 3,35 *niedriger* als der zum Budgetausgleich notwendige Beitragssatz im Kollektiv der SPV-Versicherten. Der Beitragssatz läge damit bei weniger als 30 % des Beitragssatzes in der sozialen Pflegeversicherung.

Die beiden Versichertenkollektive unterscheiden sich in Bezug auf Einkommen und Pflegerisiko somit insgesamt um den Faktor 3,35. Von der vom Bundesverfassungsgericht geforderten „ausgewogenen Lastenverteilung“ kann also nicht die Rede sein. Vielmehr zeigt sich hier aus Gerechtigkeitsüberlegungen ein deutlicher und dringender Reformbedarf.

### Reformoptionen.

Die Herstellung einer ausgewogenen Lastenverteilung kann dadurch erfolgen, dass die gesamte Bevölkerung in *ein *System integriert wird und die Pflegevolksversicherung so nicht mehr in 2, sondern in einem Zweig realisiert wird. Entsprechende Vorschläge sind unter der Überschrift „Pflegebürgerversicherung“ schon seit vielen Jahre vorgetragen (vgl. z. B [16]) und diskutiert worden [17–19]. Eine solche integrierte Pflegeversicherung würde nicht nur die eklatante Ungleichbehandlung von Privat- und Sozialversicherten aufheben, sondern gleichzeitig dazu beitragen, den Beitragssatz in der Sozialversicherung zu stabilisieren. Nach Berechnungen für das Jahr 2017 würde eine Pflegebürgerversicherung bei Beitragspflicht für alle Einkünfte und bei einer Beitragsbemessungsgrenze in Höhe der für die Rentenversicherung (West) geltenden Höhe den zum Budgetausgleich notwendigen Beitragssatz initial um 0,5 Beitragssatzpunkte reduzieren. Im Zeitverlauf würde der differenzielle Effekt bis 2060 sogar auf knapp 0,7 Beitragssatzpunkte ansteigen [11].

Wird aus politischen Gründen eine solche integrierte Pflegevolksversicherung nicht für möglich erachtet, kann versucht werden, die Effekte der Risikoselektion durch einen Finanzausgleich zu begrenzen. Ein derartiger Finanzausgleich, der analog zu dem in der gesetzlichen Krankenversicherung praktizierten Risikostrukturausgleich ausgestaltet werden könnte, ist tatsächlich bereits einmal in einem Koalitionsvertrag vereinbart worden, nämlich 2005 im Koalitionsvertrag zwischen CDU/CSU und SPD, dann aber nicht umgesetzt worden. Bei entsprechender Ausgestaltung kann er bezüglich der derzeitigen Ungleichbehandlung der Versicherten als funktionales Äquivalent zu einer integrierten Versicherung dienen. Allerdings würden bestehende Doppeltstrukturen in der Verwaltung der Pflegeversicherten dabei perpetuiert, so dass diese Lösung aus Effizienzgesichtspunkten unterlegen ist.

## Trennung von Kranken- und Pflegeversicherung

Alternativ zur später realisierten Version einer eigenständigen Pflegeversicherung wurden im Vorfeld verschiedene Reformoptionen diskutiert, u. a. ein Bundesleistungsgesetz und die Integration von Leistungen bei Pflegebedürftigkeit in die gesetzliche Krankenversicherung [20]. Tatsächlich wurden im Gesundheits-Reformgesetz (GRG) vom 29.12.1989 (BGBl. I, S. 2477) erstmals „Leistungen bei Schwerpflegebedürftigkeit“ in die GKV integriert (§§ 53–57 SGB V idF des GRG). Seitdem wird darüber diskutiert, ob die Einführung einer eigenen Säule der Sozialversicherung tatsächlich die überlegene Lösung war und ob eine Zusammenführung der Kranken- und Pflegeversicherung sinnvoll sei (vgl. [21]).

### Bewertungsmaßstab.

Begründet wird der Vorstoß zur Zusammenlegung regelmäßig mit Schnittstellenproblemen im Verhältnis von Kranken- und Pflegeversicherung, die eine optimale medizinische und pflegerische Versorgung pflegebedürftiger Menschen verhindere, und die es durch die Systemintegration zu lösen gelte. Damit ist ein sinnvoller Bewertungsmaßstab benannt: die reibungslose Gewährleistung des Ineinandergreifens verschiedener Versorgungsleistungen. Gleichzeitig ist aber zu beachten, dass sich das Pflegeverständnis in den letzten Dekaden deutlich verändert hat. Neben Versorgungsleistungen im engeren Sinne sollen die Leistungen der Sozialversicherung darauf hinwirken, die gesellschaftliche Teilhabe pflegebedürftiger Menschen sicherzustellen. Auch dies ist bei der Bewertung verschiedener Ausgestaltungsoptionen zu beachten.

### Problemlage.

Probleme zeigen sich an der Schnittstelle von Kranken- und Pflegeversicherung vor allem an 2 Stellen: der Rehabilitation und der medizinischen Behandlungspflege. Pflegeversicherte haben einen Rechtsanspruch „gegen den zuständigen Träger auf Leistungen zur medizinischen Rehabilitation“, wenn diese „zur Beseitigung, Minderung oder Verhütung einer Verschlimmerung der Pflegebedürftigkeit … geeignet, notwendig und zumutbar sind“ (§ 18 Abs. 1 Satz 3 SGB XI). Dieser Rechtsanspruch ist im Pflege-Weiterentwicklungsgesetz vom 28.05.2008 (BGBl I, S. 874) sogar noch ausgeweitet worden: Bezog er sich vorher auf „Leistungen zur ambulanten Rehabilitation mit Ausnahm von Kuren“, bezieht er sich nunmehr auf *jegliche Form* der „medizinischen Rehabilitation“. Zuständiger Träger ist bei Versicherten im Rentenalter regelmäßig die Krankenkasse. Allerdings stehen die Krankenkassen als Träger der GKV miteinander im Wettbewerb. Leistungsausgaben für Rehabilitation belasten die Kassen daher und erhöhen – unter sonst gleichen Bedingungen – ihren notwendigen (Zusatz‑)Beitragssatz und sind deshalb nur attraktiv, wenn sie zu Einsparungen in anderen Leistungsbereichen führen, die ebenfalls beitragswirksam sind. Aufgrund des vollständigen Ausgabenausgleichs in der Pflegeversicherung sind Rehabilitationsmaßnahmen, die lediglich auf die „Beseitigung, Minderung oder Verhütung einer Verschlimmerung der Pflegebedürftigkeit“ abzielen, aber gerade *nicht* beitragssatzwirksam, da die Pflegeversicherung als „Einheitskasse“ konzipiert ist (vgl. hierzu bereits [22]). Für wettbewerblich denkende Kassen „lohnt“ sich Rehabilitation zur Beeinflussung von Pflegebedürftigkeit nicht, da die Rehabilitation aus der – wettbewerblich organisierten – Krankenversicherung finanziert wird, die Erfolge aber die – nicht wettbewerblich organisierte – Pflegeversicherung entlasten. Deren Ausgabenreduktionen werden aber über den allgemeinen Ausgabenausgleich der Pflegeversicherung an alle Pflegekassen verteilt, während die Kosten der Rehabilitation nur die jeweilige Krankenkasse belasten und deren Wettbewerbsposition im Verhältnis zu anderen Kassen verschlechtern. Kassen haben damit einen objektiven Anreiz, Ausgaben für Rehabilitation zu begrenzen.

Die Kosten der *medizinischen Behandlungspflege* im Pflegeheim gelten als mit dem Pflegesatz abgedeckt. Da die als Zuschüsse ausgestalteten Leistungen der Pflegeversicherung bei vollstationärer Pflege aber deutlich unterhalb der in Rechnung gestellten Entgelte für pflegebedingte Aufwendungen liegen, werden die Kosten der medizinischen Behandlungspflege de facto von den Pflegebedürftigen über ihren Eigenanteil selbst finanziert. Die Ausgaben für das funktionale Äquivalent zur medizinischen Behandlungspflege im ambulanten Bereich, nämlich die häusliche Krankenpflege nach § 37 SGB V, werden hingegen von der Krankenkasse übernommen und sind damit wettbewerbsrelevant. Wird die pflegebedürftige Person stationär versorgt, vermeidet die Kasse diese Ausgaben. Angesichts der Gesamtausgaben der GKV für häusliche Krankenpflege, die sich inzwischen – bei stark zunehmender Tendenz – auf mehr als 9 Mrd. € pro Jahr (für 2021) belaufen, und geschätzter Kosten der medizinischen Behandlungspflege in Pflegeheimen von 2–3 Mrd. € ist dieser Ausgabenposten auch quantitativ nicht zu vernachlässigen. Die Kassen haben somit einen objektiven Anreiz – entgegen dem Gesetzesauftrag „ambulant vor stationär“ (§ 3 SGB XI) – stationäre Pflege zu präferieren und Pflegebedürftige entsprechend zu beraten.

### Reformoptionen.

Die weitestgehende Reform zur Behebung der genannten Fehlanreize wäre die Zusammenlegung von Kranken- und Pflegeversicherungen. Allerdings greift es zu kurz, Pflegebedürftige lediglich als multimorbide Menschen mit komplexen Versorgungsbedarfen zu betrachten. Vielmehr müssen Leistungen bei Pflegebedürftigkeit auf den Erhalt der vorhandenen Kompetenzen abzielen – ein Aspekt, der im Medizinsystem nur eine untergeordnete Rolle spielt. Zentral ist zudem die Sicherung der sozialen Teilhabe durch entsprechende Leistungen. Schließlich werden Sorgeleistungen vor allem von pflegenden An- und Zugehörigen erbracht, die durch Leistungen bei Pflegedürftigkeit unterstützt werden sollen. Bei einer Integration der Pflegeversicherungsleistungen in die Krankenversicherung – und angesichts der Größenverhältnisse der beiden Systeme kann eine Integration nur in diese Richtung erfolgen – besteht die Gefahr, dass diese Besonderheiten der Langzeitpflege nicht hinreichend beachtet würden. Stattdessen wäre eine Medikalisierung der Langzeitpflege zu erwarten, die die Rolle der Pflege in der Versorgung Pflegebedürftiger zugunsten der Medizin abwerten würde (so auch [23]). Insofern erscheint die Strukturentscheidung für ein eigenständiges System auch rückblickend als sinnvoll, da sie die Etablierung der Langzeitpflege als eigenes allgemeines Lebensrisiko mit spezifischen Versorgungs- und Unterstützungsbedarfen entscheidend gefördert hat und auch heute noch fördert.

Sinnvoll ist es allerdings, die genannten Fehlanreize gezielt im Rahmen des gegliederten Systems anzugehen. Die fehlenden Anreize zur Rehabilitation wurden vom Gesetzgeber im Pflege-Weiterentwicklungsgesetz vom 28.05.2008 (BGBl. I S. 874) aufgegriffen. In diesem Gesetz wurden die *Kranken*kassen verpflichtet, eine „Strafzahlung“ an die jeweilige *Pflege*kasse zu leisten, sollten sie durch eigenes Verschulden eine von der Pflegeversicherung als notwendig angesehene medizinische Rehabilitation nicht erbracht haben (§ 40 Abs. 3 SGB V). Dabei hat der Gesetzgeber unterstellt, dass zwischen einer Krankenkasse und „ihrer“ Pflegekasse ein Interessenkonflikt besteht. Tatsächlich sind die Mitarbeiter:innen der Kranken- und der zugehörigen Pflegekasse aber Teil *eines *Unternehmens. Tatsächlich ist es nicht so, dass sich „die Krankenversicherung“ auf Kosten „der Pflegeversicherung“ entlastet (funktionale Betrachtung), sondern dass „Kassen“ – als Träger von Kranken- und Pflegeversicherung (institutionelle Betrachtung) – ihre Wettbewerbsposition gegenüber anderen Kassen verbessern, indem sie die Kosten von der Abteilung Krankenversicherung in die Abteilung Pflegeversicherung verschieben bzw. eine Rehabilitationsmaßnahme unterlassen, die Kosten in der Abteilung Pflegeversicherung reduziert, dafür aber Kosten in der Abteilung Krankenversicherung verursacht. Die gesetzliche Maßnahme hat sich daher als untauglich erwiesen [24].

Zielführender wäre dagegen die *Übertragung der Finanzierungskompetenz* für die geriatrische Rehabilitation von der Kranken- in die Pflegeversicherung, also in das System, in dem auch der Nutzen einer Rehabilitation „zur Beseitigung, Minderung oder Verhütung einer Verschlimmerung der Pflegebedürftigkeit“ anfällt. Da die Ausgaben für Pflegeleistungen für die Kassen nicht wettbewerbsrelevant sind, entstehen damit zwar keine starken Anreize zur Förderung von Rehabilitation, aber zumindest werden die Anreize *gegen* die Gewährung von Maßnahmen der Rehabilitation abgebaut.

Auch bezüglich der medizinischen Behandlungspflege würden die Fehlanreize abgebaut, wenn die Finanzierungsverantwortung für medizinische Behandlungspflege wieder an die Krankenversicherung überginge (vgl. [25]). Eine solche Neuordnung der Finanzierungskompetenz entspräche zudem der Grundlogik, nach der die GKV und nicht die Pflegeversicherung für die medizinische Versorgung zuständig ist [23]. Tatsächlich sieht der aktuelle Koalitionsvertrag vor, „die Behandlungspflege in der stationären Versorgung der gesetzlichen Krankenversicherung [zu] übertragen und pauschal aus[zu]gleichen“ [8, S. 80 f.]. Allerdings sind dieser Ankündigung ein Jahr nach Abschluss des Koalitionsvertrags noch keine Taten gefolgt.

## Fazit

Die Pflegeversicherung kann als ein Beispiel für pfadabhängige Entwicklungen angesehen werden, bei der vergangene Entscheidungen die Entwicklung auf einen „Pfad“ vorfestlegen, der nur schwer wieder verlassen werden kann. So prägen die bei ihrer Einführung getroffenen Grundsatzentscheidungen die Pflegeversicherung bis heute. Dies gilt insbesondere für die benannten Geburtsfehler, die bis heute nicht behoben werden konnten. Während eine Neuaufteilung der Finanzierungskompetenz für Rehabilitation und die medizinische Behandlungspflege in der nächsten Reform als möglich erscheinen, ist die Einführung eines aus Gerechtigkeitsgründen angezeigten Finanzausgleichs zwischen sozialer Pflegeversicherung und privater Pflegepflichtversicherung derzeit wenig wahrscheinlich – obwohl ein solcher Finanzausgleich bereits einmal vereinbart war, im Koalitionsvertrag zwischen CDU/CSU und SPD im Jahr 2005. Auch bezüglich der Eigenanteile scheint der Politik die Kraft für eine radikale, das Problem an der Wurzel packende Lösung zu fehlen. Obwohl SPD, Grüne und Linke eine absolute Begrenzung der Eigenanteile im Wahlprogramm zur letzten Bundestagswahl hatten und auch der Kanzlerkandidat der CDU/CSU eine absolute Begrenzung der pflegebedingten Eigenanteile auf monatlich 700 € in sein „100-Tage-Programm“ vom September 2021 aufgenommen hatte, stehen die Chancen für eine derartige Reform derzeit schlecht. Vielmehr sieht der Referentenentwurf zum Pflegeunterstützungs- und -entlastungsgesetz lediglich vor, die Leistungszuschläge zu den Eigenanteilen nach § 43c SGB XI leicht anzuheben. Dies würde zwar zu einer kurzfristigen Entlastung bei den Eigenanteilen führen, diese würden aber schnell wieder von steigenden Pflegesätzen kompensiert wird, so dass sich die Problematik am Ende der Legislaturperiode wieder genauso darstellen wird wie derzeit.

## Footnotes

1 https://www.pro-pflegereform.de/; letzter Zugriff: 26.03.2023.
